# Avoiding concentration quenching and self-absorption in Cs_4_EuX_6_ (X = Br, I) by Sm^2+^ doping

**DOI:** 10.1039/d2tc05311j

**Published:** 2023-01-27

**Authors:** Casper van Aarle, Karl W. Krämer, Pieter Dorenbos

**Affiliations:** a Faculty of Applied Sciences, Delft University of Technology Mekelweg 15 Delft The Netherlands c.vanaarle@tudelft.nl; b Department of Chemistry and Biochemistry, University of Bern Freiestrasse 3 Bern Switzerland

## Abstract

The benefits of doping Cs_4_EuBr_6_ and Cs_4_EuI_6_ with Sm^2+^ are studied for near-infrared scintillator applications. It is shown that undoped Cs_4_EuI_6_ suffers from a high probability of self-absorption, which is almost completely absent in Cs_4_EuI_6_:2% Sm. Sm^2+^ doping is also used to gain insight in the migration rate of Eu^2+^ excitations in Cs_4_EuBr_6_ and Cs_4_EuI_6_, which shows that concentration quenching is weak, but still significant in the undoped compounds. Both self-absorption and concentration quenching are linked to the spectral overlap of the Eu^2+^ excitation and emission spectra which were studied between 10 K and 300 K. The scintillation characteristics of Cs_4_EuI_6_:2% Sm is compared to that of the undoped samples. An improvement of energy resolution from 11% to 7.5% is found upon doping Cs_4_EuI_6_ with 2% Sm and the scintillation decay time shortens from 4.8 s to 3.5 s in samples of around 3 mm in size.

## Abstract 2

Cs_4_EuX_6_ scintillators suffer from strong self-absorption and weak concentration quenching. Both can be avoided by Sm^2+^ doping. It improves the energy resolution, shortens the decay time and shifts the emission wavelength into the near-infrared.

## Graphical abstract

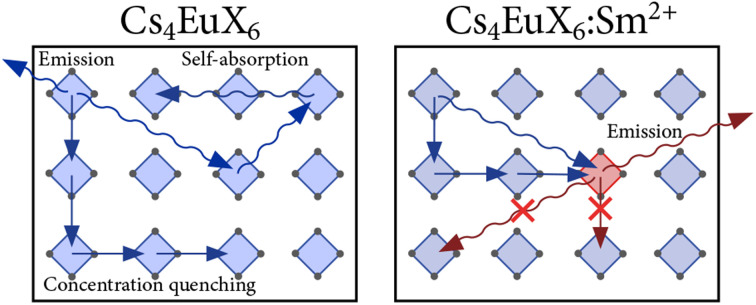

## 1. Introduction

The energy resolution is a key parameter in the development of γ-ray spectrometers. It is defined as the full width at half maximum of the photopeak in a pulse height spectrum and is a measure of how accurately γ-rays from different energies can be resolved. Typically, scintillator energy resolutions are compared under detection of 662 keV γ-rays from a ^137^Cs source. The current best energy resolution of 2.04% was achieved with LaBr_3_:Ce,Sr, for which a light yield of 70 000 ph per MeV was reported.^1^ The energy resolution of LaBr_3_:Ce,Sr is close to the fundamental limit based on photon statistics, meaning significant improvements can only be made if more photons are detected in a scintillation event. This stresses the importance of developing high light yield scintillators. After the rediscovery of SrI_2_:Eu in 2008,^2^ SrI_2_:Eu has been further developed resulting in an energy resolution of 2.6% and light yields of up to 115 000 ph per MeV have been reported.^3–7^ Among other Eu^2+^-doped halides, the best energy resolution of 2.3% was achieved with CsBa_2_I_5_:Eu, with reported light yields up to 100 000 ph per MeV.^8–11^ This shows that Eu^2+^-doped halides are potential candidates for achieving an energy resolution below 2%.

Despite these promising characteristics, Eu^2+^-doped scintillators still suffer from several drawbacks. Light yield and energy resolution typically tend to improve upon initial increase in Eu^2+^ concentration, but above several percent doping with Eu^2+^ these properties start to worsen again.^12–16^ This is at least in part caused by the large overlap between the Eu^2+^ excitation and emission spectrum, making Eu^2+^-doped halides prone to concentration quenching and, in the case of large crystals, self-absorption.^3,17,18^

Concentration quenching is caused by energy transfer between Eu^2+^ ions. This can take the form of consecutive transfer of a single excitation from one Eu^2+^ to another until it reaches a quenching site, or the excitation energy being lost by transfer to an already excited Eu^2+^ ion after which energy is lost due to relaxation back to the emitting state.^19,20^ Both these processes are strongly dependent on distance between Eu^2+^ ions and their effects can thus be diminished by increasing the distance between Eu^2+^ ions.^21^ Aside from reducing the amount of Eu^2+^ doping, this can also be achieved by selecting host compounds which intrinsically have larger distances between the divalent cation sites.

The compounds Cs_4_MX_6_ (M = Ca, Sr, Eu, Yb; X = Br, I) crystallize in the K_4_CdCl_6_-type structure with space group *R*3̄*c*.^22^ They contain a single M^2+^ site with isolated [MX_6_]^4−^ octahedra, well separated from each other by Cs^+^ cations. Accordingly, the smallest M–M distance of *R* = 9.0 Å in Cs_4_EuI_6_^22–24^ is much longer than for corner-sharing perovkites with *R* = 6.2 Å in CsEuI_3_.^25^ When only considering the dipole–dipole interaction (scaling with *R*^−6^ ^21^), the expected rate of energy transfer between Eu^2+^ nearest neighbours is around 10 times slower in Cs_4_EuI_6_ than it is in CsEuI_3_. Contribution of the exchange interaction will make this difference even larger. Based on this, one would expect that Cs_4_MX_6_ compounds are much less affected by concentration quenching than their CsMX_3_ counterparts, which is in line with the light yield increase reported by Wu *et al.* upon lowering the dimensionality of self-activated Cs_*n*_EuI_2+*n*_ compounds.^24^

Even though concentration quenching can be reduced by careful selection of the host lattice, the problem of self-absorption remains, especially for high Eu^2+^ concentration and large crystal size. A solution of the Eu^2+^ self-absorption problem in scintillators is the co-doping with Sm^2+^. With an addition of as little as 1% Sm^2+^, close to 100% of Eu^2+^ excitations are transferred to Sm^2+^ and almost exclusively Sm^2+^ emission is observed.^26,27^ The Sm^2+^ 4f^5^5d → 4f^6^ emission can end up in any of the 4f^6^ [^7^F_*J*_] states, while re-absorption can only take place from the 4f^6^ [^7^F_0_] ground state. Transitions to the 4f^6^ [^7^F_1−6_] states result in longer wavelength emissions that are less likely to be re-absorbed by other Sm^2+^ ions. In combination with the relatively low Sm^2+^ concentration of around 1%, the probably of self-absorption in Eu^2+^ and Sm^2+^ co-doped materials is greatly reduced compared to scintillators in which Eu^2+^ is the emitting ion.^28^

In this work, the benefits of doping Cs_4_EuBr_6_ and Cs_4_EuI_6_ with Sm^2+^ are studied in an attempt to solve self-absorption and concentration quenching and thereby develop a near-infrared scintillator. For this, Cs_4_EuBr_6_ and Cs_4_EuI_6_ samples were synthesizes with Sm^2+^ concentrations ranging from 0% to 2%. The amount of self-absorption is assessed for undoped and Sm^2+^-doped Cs_4_EuI_6_ through X-ray excited emission and decay measurements. Energy transfer from Eu^2+^ to Eu^2+^ and from Eu^2+^ to Sm^2+^ is studied through photoluminescence spectroscopy and decay studies. Lastly, the scintillation performance is assessed through ^137^Cs 662 keV γ-ray excited pulse height spectra.

## 2. Experimental techniques

Crystals of Cs_4_EuBr_6_, Cs_4_EuI_6_, and doped crystals with 0.5% and 2% Sm^2+^ were grown from the binary halides by the vertical Bridgman technique. CsBr (5N, Alfa) and CsI (suprapur, Merck) were dried in high vacuum at 200. EuBr_2_ and SmBr_3_ were prepared by the ammonium bromide method.^29^ The rare earth oxide M_2_O_3_ (M = Eu, 5N, Metall Rare earth Ltd; M = Sm, >3N, Fluka) was dissolved in concentrated HBr acid (suprapur, Merck) and an excess of NH_4_ Br (pro analysis, sublimed, Merck) added in a M to NH_4_ ratio of 2 to 7. The solution was dried up on a sand bath to yield the anhydrous ternary compound (NH_4_)_3_MBr_6_, which is subsequently decomposed to the binary bromide by heating in vacuum. EuBr_2_ was obtained at 500 °C and used without further purification. SmBr_3_ was sublimed at 650 °C in a silica apparatus under high vacuum for removal of SmOBr traces. SmBr_2_ was obtained by reduction of SmBr_3_ with Sm metal (3N; Alfa) in a Ta ampoule. The Ta-ampoule was sealed by helium arc-welding and enclosed into a silica ampoule under vacuum. The ampoule was heated to 900 °C for 7 days. The rare earth iodides were synthesized from the elements (Eu, 3N; Sm, 3N, both Alfa; I_2_, pro analysis, sublimed, Merck) in sealed silica ampoules under vacuum. EuI_2_ was obtained at 500 °C and purified by sublimation in an Au ampoule under vacuum at 900 °C. SmI_3_ was prepared at 700 °C and sublimed for purification at 800 °C. SmI_2_ was prepared from SmI_3_ and Sm in a Ta ampoule 1 day at 900 °C and 7 days at 600 °C.

Stoichiometric amounts of the binary halides (about 5 g per sample) were sealed in Ta ampoules, as described above. The ampoules were heated in a Bridgman furnace to 560 °C, *i.e.*, above the congruent melting points of Cs_4_EuBr_6_ at 545 °C and Cs_4_EuI_6_ at 540 °C. After 1 day at constant temperature, the crystal growth was started by slowly moving up the furnace. The samples were cooled to RT within about 10 days. Crystals were cleaved from the boules for spectroscopic investigations. The denoted doping level represents the melt composition. Since staring materials and products are highly hygroscopic and sensitive to oxidation, all handling was done under strictly dry and oxygen-free conditions (H_2_O and O_2_ < 0.1 ppm) in glove boxes and sealed sample containers.

X-ray excited emission spectra were measured using an X-ray tube with tungsten anode, operated at 79 kV and aluminium filter to block low energy X-rays for preventing radiation damage at the sample surface. The samples were attached to the cold finger of a Janis VPF-700 cryostat. The emission coming from the sample under a 90 angle with respect to the X-ray beam was coupled into an optical fibre and read out with an Ocean insights QE Pro spectrometer.

X-ray excited decay curves were measured using a time correlated single photon counting method. A start signal was generated upon triggering a PicoQuant LDH-P-C-400M laser diode exciting a Hamamatsu N5084 light excited X-ray tube with tungsten anode operated at 40 kV, creating a 500 ps long pulse of X-rays. Upon detection of a scintillation photon by the ID Quantique ID100-50 single-photon detector, a stop signal was generated. The start and stop signals were processed using an Ortec 567 time-to-amplitude converter of which the output was connected to an Ortec AD114 16k analog-to-digital converter. The sample was attached to the cold finger of a Janis VPF-700 cryostat. The sample chamber was kept at a vacuum of 10^−5^ mbar to protect the hygroscopic sample from moisture.

^137^Cs 662 keV γ-ray pulse height spectra of undoped samples were recorded using a R1791 photomultiplier tube (PMT) operated at a voltage of −700 V. The unpolished bare crystal was placed on the entrance window of the PMT and was covered with Teflon tape. No optical coupling was used. The number of photoelectrons created in a scintillation event was determined by comparing corresponding channel with the single electron response of the PMT. The light yield was calculated using the number of photoelectrons at the maximum of the 662 keV photopeak and correcting for the quantum efficiency and reflectivity of the PMT, using the method described by de Haas and Dorenbos.^30^

^137^Cs excited pulse height spectra of Sm^2+^-doped samples were recorded using an Advanced Photonix APD (type 630-70-72-510) operated at a bias voltage of 1690 V. The temperature of the APD was stabilised at 260 K. The APD signal was increased by a Cremit CR-112 pre-amplifier. The rest of the electronics are the same as used in the PMT set-up described above. The light yield was determined by comparing the channel of the photopeak with the peak from direct detection of 17.8 keV X-rays of ^241^Am.

Photoluminescence excitation and emission spectra were measured using a 450 W Xenon lamp and a Horiba Gemini 180 monochromator to excite the sample. Light from the sample was collected at a 90 angle with respect to the incoming excitation light. Reflected excitation light was filtered out with an optical filter. The emission light passed through a Princeton Instruments SpectraPro-SP2358 monochromator, after which it was detected by a Hamamatsu R7600-20 PMT. The sample was attached to the cold finger of a closed cycle helium cryostat.

Photoluminescence decay curves were recorded using an EKSPLA NT230 OPO laser to excite the sample with a repetition rate of 100 Hz and pulse duration of 10 ns. The emission passed through a Princeton Instruments SpectraPro-SP2358 monochromator and was detected by a Hamamatsu R7600U-20 PMT. An optical long pass filter was placed at the entrance of the monochromator to filter out the excitation light from the laser. The signal from the PMT was recorded using a CAEN DT5730 digitizer.

## 3. Results

**Fig. 1:**
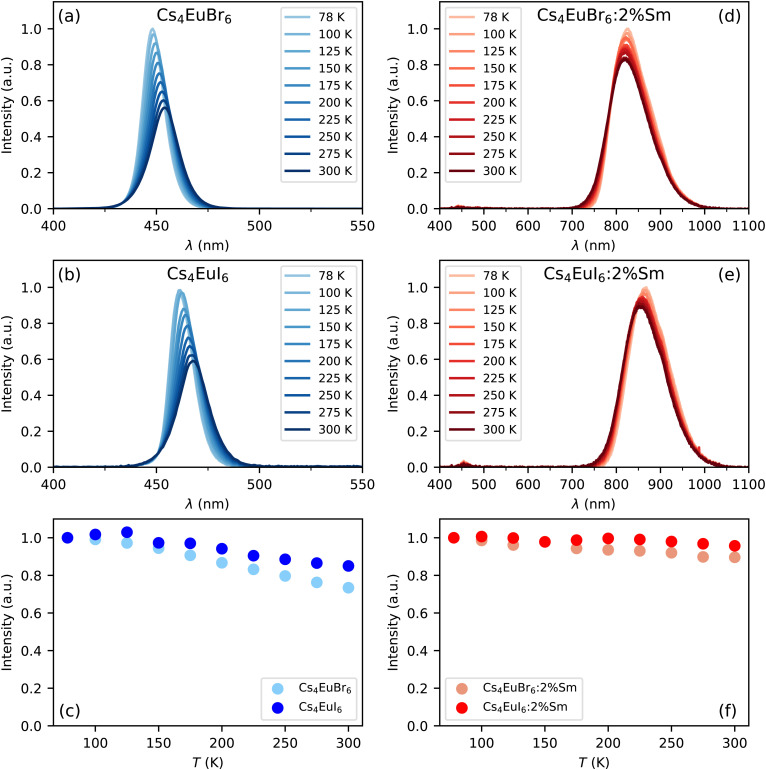
X-ray excited emission spectra between 78 K and 300 K for (a) Cs_4_EuBr_6_, (b) Cs_4_EuI_6_, (d) Cs_4_EuBr_6_:2% Sm and (e) Cs_4_EuI_6_:2% Sm. (c) and (f) show the integrated emission intensities normalised to the intensity at 78 K.

Fig. 1a and b show the X-ray excited emission spectra between 78 K and 300 K of undoped Cs_4_EuBr_6_ and Cs_4_EuI_6_, respectively. Cs_4_EuBr_6_ shows a single emission band around 450 nm. This emission band is assigned to the Eu^2+^ 4f^6^ 5d → 4f^7^ transition. With increasing temperature, the peak of the emission band shifts to longer wavelengths. This is typically observed in materials with high Eu^2+^ concentration and is ascribed to self-absorption.^8,13,31^ Cs_4_EuI_6_ also shows a single emission band that is assigned to the Eu^2+^ 4f^6^ 5d → 4f^7^ transition. The Eu^2+^ emission is located around 470 nm, at about 20 nm longer wavelength than for Cs_4_EuBr_6_. The emission band shows a similar shift to longer wavelengths with increasing temperature as was observed for Cs_4_EuBr_6_. The room temperature emission spectra of both samples correspond well to the data reported by Wu *et al.*^24^Fig. 1c shows the integrated emission intensity for Cs_4_EuBr_6_ and Cs_4_EuI_6_, normalised to their intensity at 78 K. The emission intensity of Cs_4_EuBr_6_ decreases by about 25% when temperature is increased from 78 K to 300 K. For Cs_4_EuI_6_, the emission intensity decreases by about 12%.

Fig. 1d and e show the X-ray excited emission spectra between 78 K and 300 K of Cs_4_EuBr_6_:2% Sm and Cs_4_EuI_6_:2% Sm, respectively. Cs_4_EuBr_6_:2% Sm shows a broad emission band around 820 nm, which is assigned to the Sm^2+^ 4f^5^5d → 4f^6^ transitions. With increasing temperature, the peak of the emission band shifts to shorter wavelengths. This is likely caused by thermal expansion of the sample, decreasing the crystal field splitting and thereby elevating the lowest 5d level to higher energies. The observation of the emission peak shifting to shorter wavelengths indicates a low probability of self-absorption of the Sm^2+^ emission. Cs_4_EuI_6_:2% Sm shows a similar broad emission band around 850 nm, which is also assigned to the Sm^2+^ 4f^5^5d → 4f^6^ transition. Also in this sample, the peak of the Sm^2+^ emission shifts to shorter wavelengths as temperature is increased. In both samples, a small amount of Eu^2+^ emission is visible between 400 nm and 500 nm. The integrated emission intensity of Cs_4_EuBr_6_:2% Sm and Cs_4_EuI_6_:2% Sm is shown in Fig. 1f. Upon increasing the temperature from 78 K to 300 K, the emission intensity remains almost constant compared to the decrease in intensity observed in the undoped samples.

**Fig. 2:**
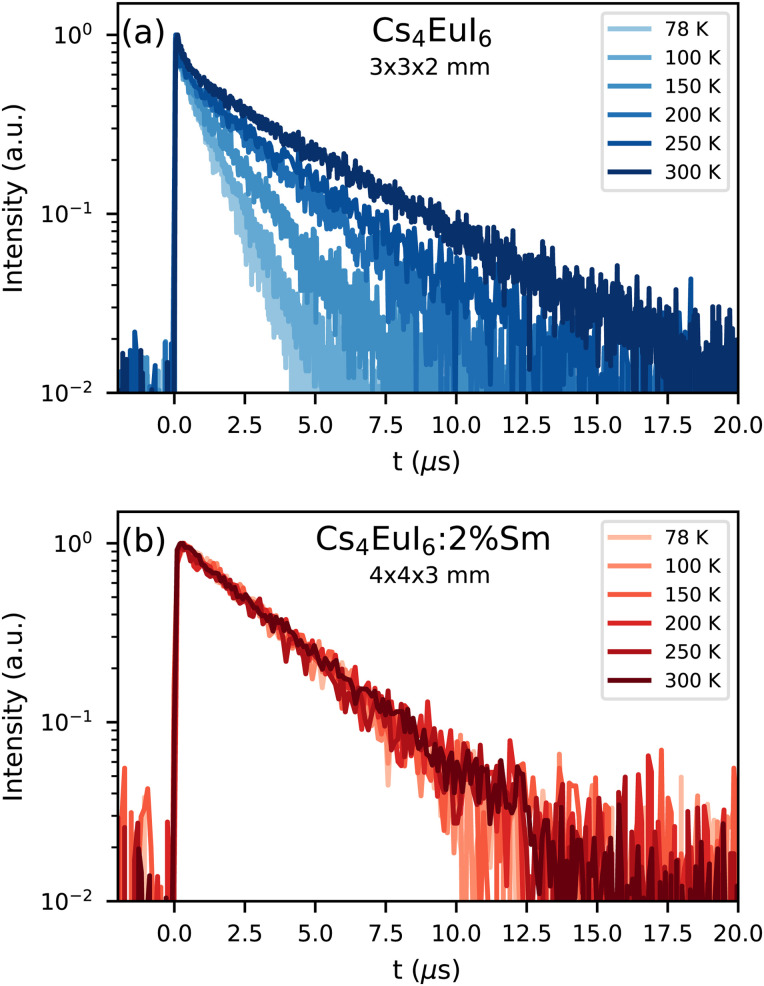
X-ray excited decay curves between 78 K and 300 K of (a) Cs_4_EuI_6_ and (b) Cs_4_EuI_6_:2% Sm.

Fig. 2a shows the X-ray excited decay curves of a 3 × 3 × 2 mm^3^ Cs_4_EuI_6_ crystal between 78 K and 300 K. All decay curves show an initial fast component after which they converge to a single exponential of which the decay time increases with temperature. At 78 K, this decay time is 1.19 s and at 300 K it becomes 4 times slower with a decay time of 4.80 s. Fig. 2b shows the X-ray excited decay curves of a 4 × 4 × 3 mm^3^ Cs_4_EuI_6_:2% Sm crystal between 78 K and 300 K. A 630 nm long pass filter was placed between the sample and the detector to remove the small amount of remaining Eu^2+^ emission. Unlike the decay curve of undoped Cs_4_EuI_6_, no fast component is observed in the decay curve. The decay time is also almost independent of temperature and increases marginally from 3.16 s at 78 K to 3.50 s at 300 K.

**Fig. 3:**
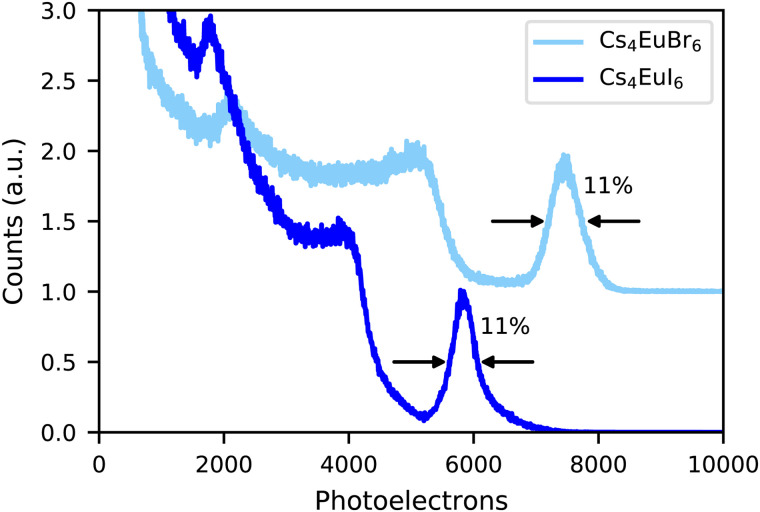
^137^Cs excited pulse height spectra of Cs_4_EuBr_6_ and Cs_4_EuI_6_ measured on a PMT.

Fig. 3 shows the ^137^Cs 662 keV γ -ray pulse height spectra of undoped Cs_4_EuBr_6_ and Cs_4_EuI_6_ recorded on a PMT. The photopeak of Cs_4_EuBr_6_ corresponds to 7500 photoelectrons being emitted from the photocathode of the PMT. Taking into account the quantum efficiency (25%) and reflectivity (33%) of the PMT, the light yield is estimated around 30 200 ph per MeV. Scintillation events in the photopeak of Cs_4_EuI_6_ correspond to 5800 photoelectrons. At its emission wavelength of 470 nm, the quantum efficiency of the PMT is 22% and the reflectivity is 35%. This gives a light yield of 26 100 ph per MeV. Both samples have an energy resolution of 11%.

**Fig. 4:**
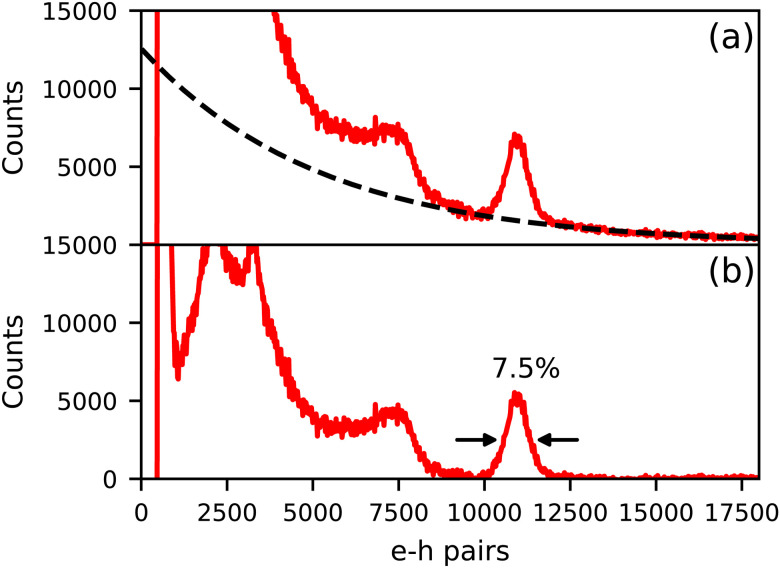
^137^Cs excited pulse height spectrum of Cs_4_EuI_6_:2% Sm measured on an APD. (a) Shows the spectrum as recorded and (b) shows the spectrum after background subtraction.

Fig. 4 shows the ^137^Cs excited pulse height spectrum of Cs_4_EuI_6_:2% Sm recorded on an APD. In Fig. 4a, the pulse height spectrum is shown as recorded. On the *x*-axis, the number of primary electron–hole pairs created in the APD during a scintillation event is set out, which is equal to the amount of detected scintillation photons. This pulse height spectrum contains a background of events that are caused by absorption of γ-rays directly in the APD. The dashed curve is an exponential approximation for this background and has been subtracted from the data to result in the pulse height spectrum displayed in Fig. 4b. On average, 11 000 photons are detected under the 662 keV photopeak, translating to a light yield of around 16 600 ph per MeV. An energy resolution of 7.5% has been attained.

**Fig. 5:**
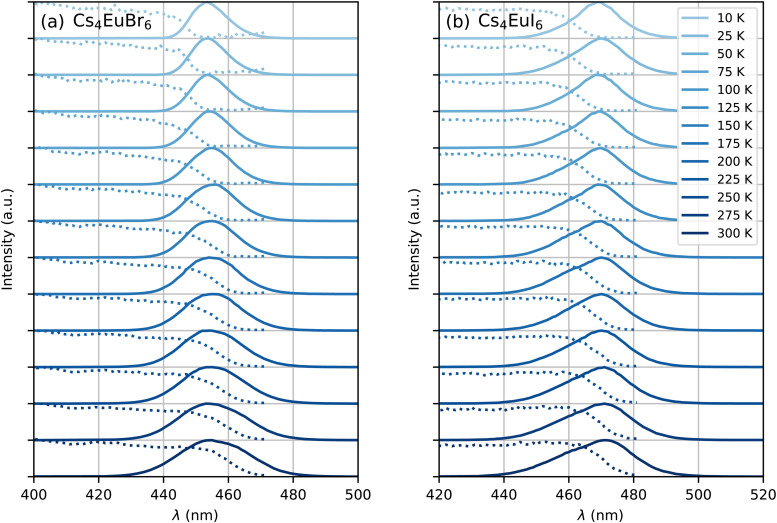
Photoluminescence excitation (dotted curve) and emission (solid curve) spectra between 10 K and 300 K of (a) Cs_4_EuBr_6_ and (b) Cs_4_EuI_6_.

Fig. 5 shows the photoluminescence excitation (dashed curves) and emission (solid curves) spectra of undoped Cs_4_EuBr_6_ and Cs_4_EuI_6_ between 10 K and 300 K. As temperature is increased, both samples show an increase in emission bandwidth and the excitation spectra stretch to longer wavelengths. The result is an increase in spectral overlap between the Eu^2+^ emission and Eu^2+^ excitation. This increase in spectral overlap increases the probability that Eu^2+^ emission is re-absorbed by other Eu^2+^, or that energy is transferred non-radiatively between neighbouring Eu^2+^ ions.

**Fig. 6:**
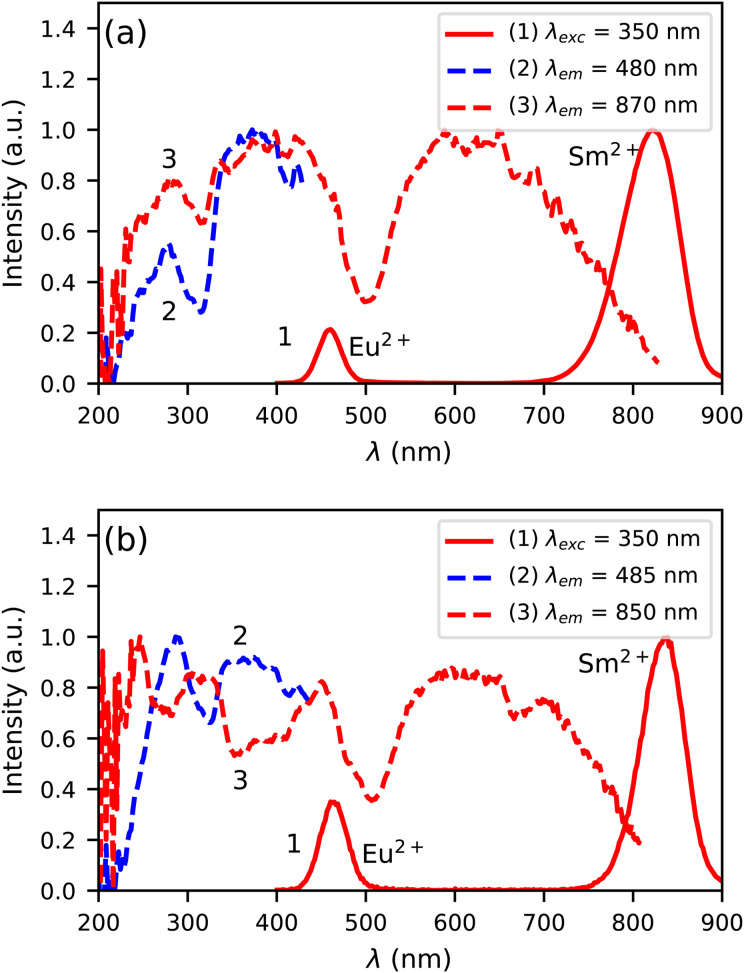
Photoluminescence excitation and emission spectra at 300 K of (a) Cs_4_EuBr_6_:0.5% Sm and (b) Cs_4_EuI_6_:0.5% Sm.

Fig. 6a shows the photoluminescence excitation and emission spectra of Cs_4_EuBr_6_:0.5% Sm at 300 K. Both the Eu^2+^ and Sm^2+^ 5d → 4f emission can be detected upon excitation at 350 nm, as shown by curve 1. The Eu^2+^ emission overlaps with the excitation spectrum of the Sm^2+^ emission (curve 3), which indicates that Eu^2+^ can transfer energy to Sm^2+^. Between 200 nm and 400 nm, the excitation spectrum of the Sm^2+^ 5d → 4f emission shows the same bands as the excitation spectrum of the Eu^2+^ 5d → 4f emission (curve 2). This confirms that energy transfer from Eu^2+^ to Sm^2+^ takes place.

In Fig. 6b, the photoluminescence excitation and emission spectra of Cs_4_EuI_6_:0.5% Sm are shown. Similar to Cs_4_EuBr_6_:0.5% Sm, it shows Eu^2+^ and Sm^2+^ 5d → 4f emission upon excitation at 350 nm, as shown by curve 1. In this case though, the excitation spectrum of the Sm^2+^ emission (curve 3) shows dips where the excitation spectrum of the Eu^2+^ emission (curve 2) shows highest intensity. This indicates that energy transfer from Eu^2+^ to Sm^2+^ is inefficient, which is likely due to the low Sm^2+^ concentration is this sample.

**Fig. 7:**
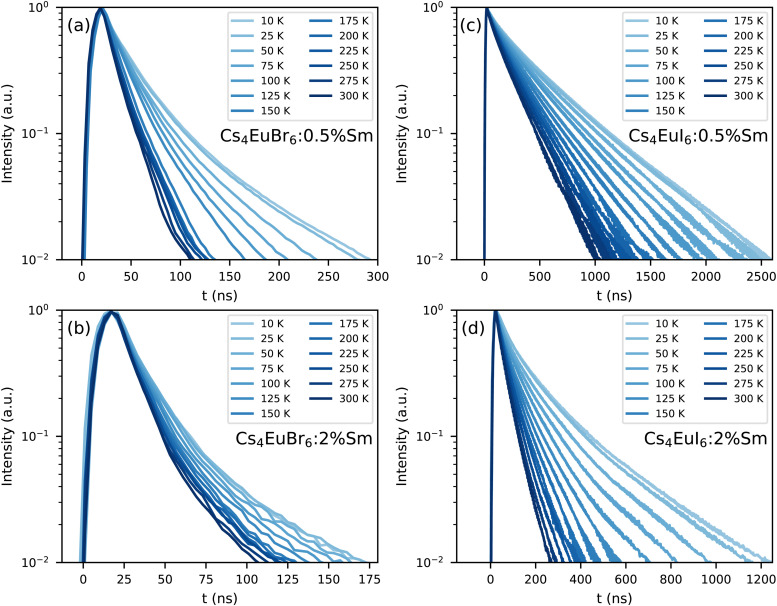
Photoluminescence decay of the Eu^2+^ emission upon 400 nm excitation between 10 K and 300 K in (a) Cs_4_EuBr_6_:0.5% Sm, (b) Cs_4_EuBr_6_:2% Sm, (c) Cs_4_EuI_6_:0.5% Sm and (d) Cs_4_EuI_6_:2% Sm.

Fig. 7 shows the photoluminescence decay of Eu^2+^ emission in (a) Cs_4_EuBr_6_:0.5% Sm, (b) Cs_4_EuBr_6_:2% Sm, (c) Cs_4_EuI_6_:0.5% Sm and (d) Cs_4_EuI_6_:2% Sm. All samples show approximately the same behaviour. All decay curves deviate from exponential functions, starting with fast decay and becoming slower as time progresses. The initial fast decay rate is only moderately dependent on temperature. The largest temperature dependence is found in the tail of the decay, making all temperature series fan out. This behaviour strongly resembles that of migrationally accelerated energy transfer, where Eu^2+^ excitations are able to move closer to Sm^2+^ by means of energy transfer between neighbouring Eu^2+^ ions.^20,32^ The initial rising component in the decay curves of Fig. 7a and b are caused by the laser pulse duration of approximately 10 ns.

The lifetime of Eu^2+^ excitations varies strongly between the samples. As the decay curves cannot be approximated with a single exponential function, the 1/e-decay time *τ*_e_ is reported. *τ*_e_ is defined as the time it takes for the decay curve to decrease by a factor of 1/e starting from its maximum intensity. Increasing the Sm^2+^ concentration in Cs_4_EuBr_6_ from 0.5% to 2% decreases *τ*_e_ from 20 ns to 16 ns. For Cs_4_EuI_6_, the same increase in Sm^2+^ concentration decreases *τ*_e_ from 172 ns to 44 ns. This decrease of *τ*_e_ with increasing Sm^2+^ concentration is expected, as a higher Sm^2+^ causes faster non-radiative energy transfer to Sm^2+^.

**Fig. 8:**
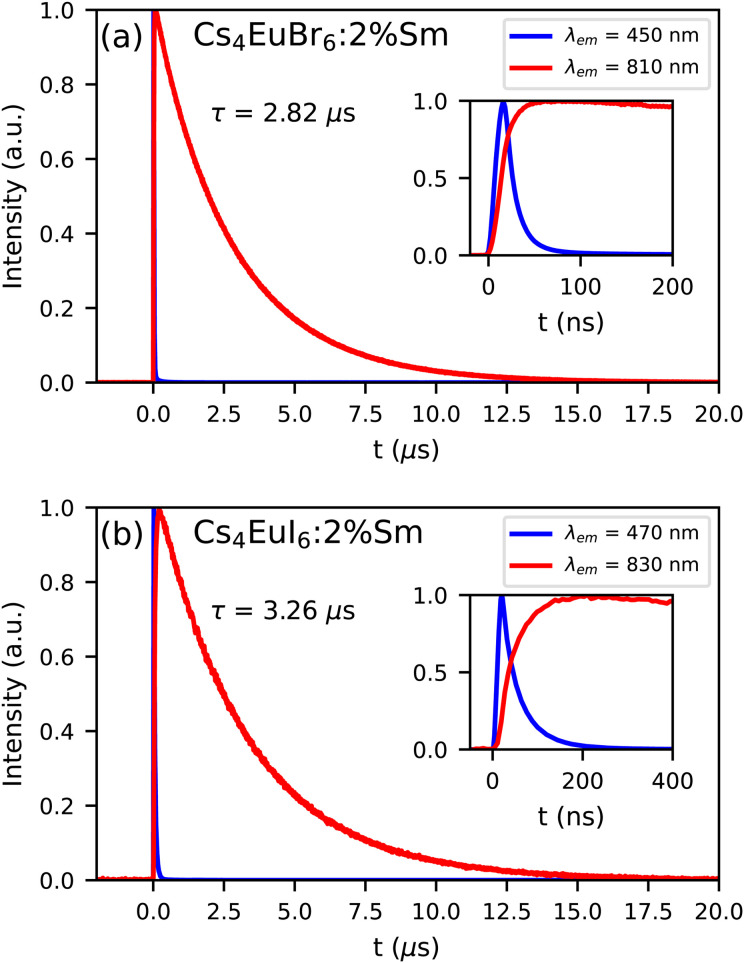
Photoluminescence decay of Eu^2+^ and Sm^2+^ emission upon 350 nm excitation at 300 K in (a) Cs_4_EuBr_6_:2% Sm and (b) Cs_4_EuI_6_:2% Sm. The insets show the same decay curve on a shorter time scale.

Fig. 8a and b show the photoluminescence decay of Eu^2+^ and Sm^2+^ emission in Cs_4_EuBr_6_:2% Sm and Cs_4_EuI_6_:2% Sm, respectively. The excitation wavelength is 350 nm, at which primarily Eu^2+^ is excited. The decay time of the Sm^2+^ emission is 2.82 s in Cs_4_EuBr_6_:2% Sm and 3.26 s in Cs_4_EuI_6_:2% Sm. The Eu^2+^ emission is strongly quenched by the presence of Sm^2+^, therefore the inset shows the start of the decay curves on a shorter timescale. Here, it becomes visible that the Sm^2+^ emission intensity increases as the Eu^2+^ emission decays. This shows that excitations are transferred from Eu^2+^ to Sm^2+^.

## 4. Discussion

Both self-absorption and non-radiative energy transfer significantly modify a luminescence decay curve. The luminescence decay can thus yield information on the energy transfer processes in the sample. The probability of both processes scales with the spectral overlap between the excitation band of the absorbing ion and the emission band of the emitting ion. In the case of Eu^2+^ it is shown by Fig. 5 that temperature induced broadening of the Eu^2+^ excitation and emission bands results in a significant change in spectral overlap between these bands when going from 10 K to 300 K. This increase in spectral overlap occurs both in Cs_4_EuBr_6_ and Cs_4_EuI_6_. Based on this, it is expected that the probability of self-absorption and the non-radiative energy transfer rate between neighbouring Eu^2+^ ions increase with temperature. Both these processes typically decrease the light yield of a scintillator, which is in line with the observed decrease of the emission intensity under X-ray excitation, as shown in Fig. 1c.

Fig. 2a shows the X-ray excited luminescence decay curves of Cs_4_EuI_6_. The experiment was performed in reflection mode, *i.e.*, the detector was pointed to the surface of the sample illuminated by X-rays. With an average X-ray energy of around 10 keV, almost all X-rays are absorbed within the first 100 m below the sample surface; therefore almost all excitations of Eu^2+^ are present close to the surface on the side of the crystal oriented towards the detector. Light emitted into the direction of the detector has a low chance of being re-absorbed, thus the fast component visible in the first few microseconds gives an impression of the intrinsic radiative decay rate of Eu^2+^. The light which, after being emitted, travels deeper into the crystal has a high probability of being re-absorbed. The absorbed photons can be re-emitted into the direction of the detector and still contribute to the decay curve. The decay curve thereby converges to an exponential decay rate that is longer than the intrinsic radiative decay rate of Eu^2+^. This decay rate is given by eqn (1)^33^:1
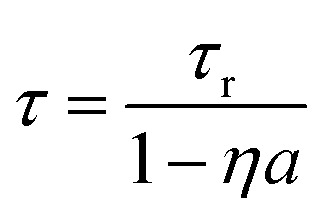
Here, *τ*_r_ is the radiative lifetime of the luminescence center, in this case Eu^2+^. *η* is the quantum efficiency of the luminescence center and *a* is the average probability that an emitted photon is re-absorbed. *a* increases with dopant concentration and size of the crystal. Values for *τ* and *a* are provided in Table 1 for the 3 × 3 × 2 mm^3^ Cs_4_EuI_6_ crystal, assuming *η* = 1. The value of *τ*_r_ was taken as 1.07 μs, which is the photoluminescence (PL) decay time at 10 K. It was assumed that self-absorption is negligible under these conditions. This assumption is justified as the photoluminescence decay time at room temperature converges to the same value of around 1.0 μs upon decreasing the Eu^2+^ concentration to nearly 0% in Cs_4_CaI_6_ and Cs_4_SrI_6_.^15^ When increasing the temperature from 78 K to 300 K, *τ* increases from 1.19 s to 4.80 s and accordingly *a* increases from 0.10 to 0.78. This shows that even in a small crystal of 3 × 3 × 2 mm^3^, 78% of the emitted photons are re-absorbed.

**Table 1:** Decay times *τ* under X-ray excitation of Cs_4_EuI_6_ and Cs_4_EuI_6_:2% Sm single crystals and the average probability *a* a scintillation photon is re-absorbed inside the crystal. The first row shows the photoluminescence (PL) decay time at 10 K, which is taken as an approximation for the radiative lifetime *τ*_r_

*T* (K)	Cs_4_EuI_6_ (3 × 3 × 2 mm^3^)		Cs_4_EuI_6_:2% Sm (4 × 4 × 3 mm^3^)	
	*τ* (μs)	*a*	*τ* (μs)	*a*
10 (PL)	1.07	—	3.12	—
78	1.19	0.10	3.16	0.01
100	1.40	0.24	3.13	0.01
150	2.08	0.49	3.34	0.06
200	2.98	0.64	3.40	0.08
250	3.97	0.73	3.31	0.06
300	4.80	0.78	3.50	0.11

In the same temperature range, the Sm^2+^ emission of the 4 × 4 × 3 mm^3^ Cs_4_EuI_6_:2% Sm sample shows only a marginal increase in *τ* from 3.16 s to 3.50 s. Using eqn (1), the value of *a* is calculated for every temperature step. The results are summarised in Table 1. Even though the Cs_4_EuI_6_:2% Sm crystal is significantly larger than the undoped Cs_4_EuI_6_ crystal, the values of *a* are around 10 times lower at every temperature. This shows that the low sensitivity of Sm^2+^ to self-absorption combined with the low Sm^2+^ concentration is an effective way to almost completely solve the self-absorption problem of Cs_4_EuI_6_.

As Eu^2+^ transfers energy to Sm^2+^, doping the samples with Sm^2+^ introduces a large amount of quenching sites for Eu^2+^. Insight in the non-radiative energy transfer rate between Eu^2+^ ions can be attained by monitoring the photoluminescence decay of Eu^2+^ in the Sm^2+^-doped samples, see Fig. 7. Directly after excitation of Eu^2+^, the Eu^2+^ excitations are randomly distributed through the lattice. As the rate of non-radiative energy transfer scales with *R*^−6^, excited Eu^2+^ ions that happen to be close to Sm^2+^ will transfer their energy to Sm^2+^ with higher probability than those at larger distance. Therefore the Eu^2+^ excitations will be depleted rapidly close to Sm^2+^. In this stage, corresponding to the first tens of nanoseconds of the decay curves, the rate at which Sm^2+^ can deplete the volume around it is the limiting factor in the decay rate of Eu^2+^. Once the volume around Sm^2+^ has been depleted, non-radiative energy transfer between Eu^2+^ ions will replenish the excitations in the depleted volumes. At this point, non-radiative energy transfer between Eu^2+^ ions becomes the limiting factor of the Eu^2+^ decay rate and causes the Eu^2+^ decay to slow down.

The rate at which Sm^2+^ depletes the Eu^2+^ excitations around it is almost independent of temperature, because the spectral overlap between the Eu^2+^ emission bands and the Sm^2+^ absorption bands does not change much with temperature. On the other hand, the rate of non-radiative energy transfer between Eu^2+^ ions does depend on temperature, because the spectral overlap between the Eu^2+^ excitation and emission spectra increases with temperature, see Fig. 5. This is also visible in Fig. 7; during the depletion of Eu^2+^ excitations close to Sm^2+^ in the first tens of nanoseconds after excitation, the Eu^2+^ emission decays with approximately the same rate at every temperature. As time further progresses into the hundreds of nanoseconds, non-radiative energy transfer between Eu^2+^ ions becomes the limiting factor of the Eu^2+^ decay; this results in a strong temperature dependence of the decay rate on longer timescales and the decay curves fan out.

The results in Fig. 7 show that a significant amount of energy transfer between Eu^2+^ ions is observed even for the isolated [MX_6_]^4−^ octahedra of Cs_4_EuX_6_. The light yield reported for undoped Cs_4_EuI_6_ (53 000 ph per MeV)^24^ is still significantly lower than that of the more diluted compounds Cs_4_CaI_6_:7%Eu (69 000 ph per MeV) and Cs_4_SrI_6_:9%Eu (78 000 ph per MeV),^15^ as reported by the same research group. When cooling Cs_4_EuI_6_ from 300 K to 78 K, the migration rate of the Eu^2+^ excitations slows down while the light yield increases 10% to 20% (Fig. 1c). These observations strongly suggest that concentration quenching still plays a role in undoped Cs_4_EuBr_6_ and Cs_4_EuI_6_.

Opposed to the undoped samples, Cs_4_EuBr_6_:2% Sm and Cs_4_EuI_6_:2% Sm show a much less significant change in intensity in the same temperature range, as shown by Fig. 1f. Once energy is transferred from Eu^2+^ to Sm^2+^ it cannot be transferred back to Eu^2+^ anymore. If the Sm^2+^ concentration is low enough, the distance between the Sm^2+^ ions is large and energy will not be transferred between Sm^2+^ ions. Aside from avoiding self-absorption, the doping of Eu^2+^ materials with Sm^2+^ effectively reduces concentration quenching.

Compared to the 2.1 s scintillation decay time of CsBa_2_I_5_:Sm^2+^, the 3.5 s decay time of Cs_4_EuI_6_:2% Sm is on the slow side for Sm^2+^-doped scintillators. However, due to the strong self-absorption in undoped Cs_4_EuI_6_, the decay time of Cs_4_EuI_6_:2% Sm is already faster than that of undoped Cs_4_EuI_6_ with sample sizes of only a few mm. It is also fast enough for applications in γ-ray spectroscopy, as successfully shown by the pulse height spectrum in Fig. 4.

## 5. Conclusions

The benefits of doping Cs_4_EuBr_6_ and Cs_4_EuI_6_ with Sm^2+^ have been studied for applications in γ-ray spectroscopy. It has been demonstrated that undoped Cs_4_EuI_6_ suffers from strong self-absorption. Despite the large distances between neighbouring Eu^2+^ ions, a small amount of concentration quenching has been observed in both Cs_4_EuBr_6_ and Cs_4_EuI_6_. Self-absorption and concentration quenching originate from the spectral overlap between the Eu^2+^ excitation and emission spectra.

Both self-absorption and concentration quenching can be avoided by doping these scintillators with 2% Sm^2+^; this results almost exclusively in Sm^2+^ 4f^5^5d → 4f^6^ emission. Due to the large amounts of self-absorption in the undoped samples, the room temperature scintillation decay time of the Sm^2+^ γ-doped Cs_4_EuI_6_ is already faster than that of small-size, undoped Cs_4_EuI_6_ crystals. The Sm^2+^ emission around 850 nm can be efficiently detected with an avalanche photodiode. Doping Cs_4_EuI_6_ with 2% Sm^2+^ improved the energy resolution from 11% to 7.5%.

## Conflicts of interest

There are no conflicts of interest to declare.

## Supplementary Material
